# Automated correction angle calculation in high tibial osteotomy planning

**DOI:** 10.1038/s41598-023-39967-w

**Published:** 2023-08-08

**Authors:** Karol Przystalski, Anna Paleczek, Karol Szustakowski, Piotr Wawryka, Michał Jungiewicz, Mateusz Zalewski, Jakub Kwiatkowski, Artur Gądek, Krzysztof Miśkowiec

**Affiliations:** 1 Medtransfer, Na Zjeździe 11, 31353 Kraków, Poland; 2 Department of Information Technologies, Jagiellonian University, Łojasiewicza 11, 30348 Kraków, Poland; 3 Faculty of Computer Science, Electronics and Telecommunications, AGH University of Science and Technology, Mickiewicza 30, 30059 Kraków, Poland; 4 Codete R &D, Na Zjeździe 11, 31353 Kraków, Poland; 5 Ortotop, Ludwinowska 11/9, 30331 Kraków, Poland; 6 Trauma and Orthopaedics Clinical Department, University Hospital in Cracow, Jakubowskiego 2, 30688 Kraków, Poland; 7 Department of Orthopedics and Physiotherapy, Jagiellonian University Medical College, Jakubowskiego 2, 30688 Kraków, Poland

**Keywords:** Bone, Cartilage, Skeleton, Computer science, Software, Scientific data

## Abstract

High tibial osteotomy correction angle calculation is a process that is usually performed manually or in a semi-automated way. The process, according to the Miniaci method, is divided into several stages to find specific points: the center of the femoral head, the edges of the tibial plateau, the Fujisawa point, the center of the ankle joint, and the Hinge point. In this paper, we proposed an end-to-end approach that consists of different techniques for finding each point. We used YOLOv4 to detect regions of interest. To identify the center of the femoral head, we used the YOLOv4 and the Hough transform. For the other points, we used a combined method of YOLOv4 with the ASM/AAM algorithm and YOLOv4 with image processing algorithms. Our fully-automated method achieved a mean error rate of 0.5$^{\circ }$ (0$^{\circ }$–2.76$^{\circ }$) ICC 0.99 (0.98–0.99) 95% CI on our own dataset of standing long-leg Anterior Posterior view X-rays. This might be the first method that automatically calculates the correction angle of high tibial osteotomy.

## Introduction

Knee osteoarthritis (OA) affects approximately 10% men and 13% women aged 60 years and older^1^. It is also one of the most common joint disorders. High tibial osteotomy (HTO) is a procedure for medial compartment osteoarthritis of the knee with varus deformity. It is a surgery where the goal is to change the load-bearing axis and reduce pain. The load change reduces cartilage degeneration by moving the weight-bearing load to the relatively unaffected lateral compartment in the varus knees. HTO was introduced many years ago in^2^. Today, it is very often the only way to prevent joint damage. Although HTO in most cases does not solve all problems, it improves long-term positive outcomes. Based on^3^, the survival rates of the patients at 5 and 10 years after HTO were 80% and 56%, respectively. The median failure time was 5.1 years. In other research^4^, only 12.8% of the patients had total knee arthroplasty after HTO and only 5.7% in the group where osteotomy was performed together with autologous cartilage implantation. Complications after surgery were observed in 8.8% of patients. Body weight and age were identified^5,6^ as the two most important factors in HTO failures. However, the performance of concomitant procedures decreased the failure rate.

There are several methods of HTO planning^7–9^. The goal of the planning is to calculate the correction angle. It consists of steps where different points are located, such as the femoral head center and knee landmarks. Up until May 2022, to the best of our knowledge, fully automatic systems for calculating the osteotomy angle using Miniaci method had not yet been implemented. There are several pieces of software available for semi-automatic calculation of the correction angle, for example, MediCAD^®^ (Hectec GmbH, Germany), PreOPlan^®^ (Siemens, Germany/Synthes, Switzerland), OrthoPilot^®^ (BBraun, Tuttlingen, Germany). These applications are used to calculate angles such as the correction angle, MPTA (medial proximal tibial angle), HKA (Hip–Knee–Ankle), etc. based on the points marked by the orthopedist. These programs also enable visualization of the leg after correction.

However, researchers have been working on the automatic detection of points such as the center of the femoral head, points within the knee joint and the ankle joint, which are then used to calculate various parameters related to the varus and valgus deformities of the lower limbs. Nguyen et al. used convolutional neural networks (CNN) to detect ten general regions of interest (ROI) containing femoral heads, knees, and ankles in complete radiographs. In the second stage, also using CNN, they detected individual points in some fragments of photos cut to previously detected ROI^10^. Schock et al. trained the UNET network to segment the limbs, then fitted a circle in the upper parts of the femurs to detect the center of the femoral head. The anatomical axes were determined using the least squares method based on the contours of the femur and tibia, and the following points were designated as binary mask stroke points. The inter-reader correlation coefficients for determination of hip–knee–ankle and anatomical-mechanical angles ranged from 0.918 to 0.995 (r range, P < 0.001), and the agreement was almost perfect (within-class correlation coefficient range 0.87–0.99)^11^. The YARLA “YOLOv4 And ResNet Landmark regression Algorithm” system was developed by Tack et al. to fully automatically find landmarks such as the center of the femoral head, lower femur, center of the upper tibia, and the talus border points necessary to determine the measures used for knee alignment, for example, HKA assessments. As the first step, they used YOLO to detect the hip joint, knee, and ankle on full leg X-rays. Then, for each assigned ROI regress landmark coordinates were detected by Residual Neural Networks (ResNet). They compared the results obtained from the calculated angles with two raters (Cooke and Duryea) of the Osteoarthritis Initiative (OAI). YARLA achieved an average mismatch of 0.09$^{\circ }$ ± 0.63$^{\circ }$ between HKA angle determinations of Cooke and Duryea. YARLA demonstrated excellent reliability measured by weighted kappa value and Spearman’s Rho value compared to both investigators. The comparison of the algorithm and the results of each rater showed a level of reliability similar to that of the comparison between the two raters^12^.

In this paper, we have introduced the automatic method to calculate the HTO correction angle according to the Miniaci method. The goal of this paper is to calculate the correction angle in high tibial osteotomy using the Miniaci method. To achieve it, we have to find a few characteristic points on the leg’s X-ray image. Our method consists of several steps: ROI detection using YOLO, and then point determination using the Hough transform, ASM and AAM as well as image processing methods. The results of our method were compared with three orthopedic specialists. We used the ICC coefficient and the Bland Altman plot analysis to evaluate the results.

## Results

**Figure 1:**
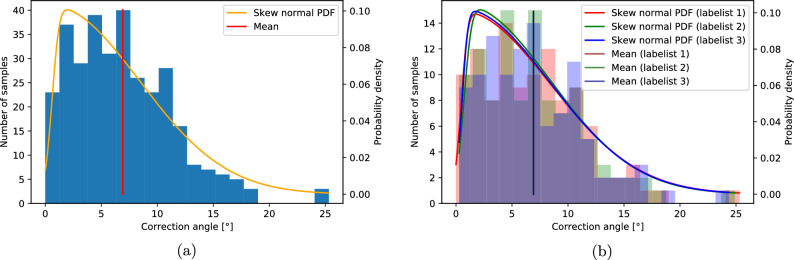
Dataset analysis. (**a**) Distribution of correction angles in the dataset. (**b**) Distribution of correction angles for each specialist separately. Values of means are close enough to overlap, forming a black line on the plot.

To get a better overview of our problem, we conducted an analysis of our data set and the results of labelling. An important aspect is the distribution of angles as labelled by the specialists. We fitted skew normal distributions of correction angles that result from labelling six points from each specialist separately, and one distribution that fits all of the correction angles in the dataset, allowing duplicate entries for a single image, since there were three specialists labelling each image. The corresponding distributions, shown in Fig. 1a, highlight some important facts about the dataset and provide ways to measure the model performance.

First, PDFs (Probability Density Functions) of angles labelled by all the specialists are similar, and the means are almost the same for each of the labelists, making it possible to compare distributions of outputs of models trained for the correction angle tasks to an unknown real distribution that can be approximated by fitting a PDF (in this case a skew-normal PDF) to angles calculated using points labelled by specialists. Even if comparison of distributions is not directly possible, at least the mean of model output should lie close to that of the total distribution shown in Fig. 1b.

Second, the range of correction angles according to the literature and orthopedic practice is 4$^\circ$–18$^\circ$. Below this range, no surgery is required and for angles greater than 18$^\circ$, surgery may not be performed^13^. The samples from our dataset contain correction angles both in the range for which the operation is suggested and outside this range.

**Figure 2:**
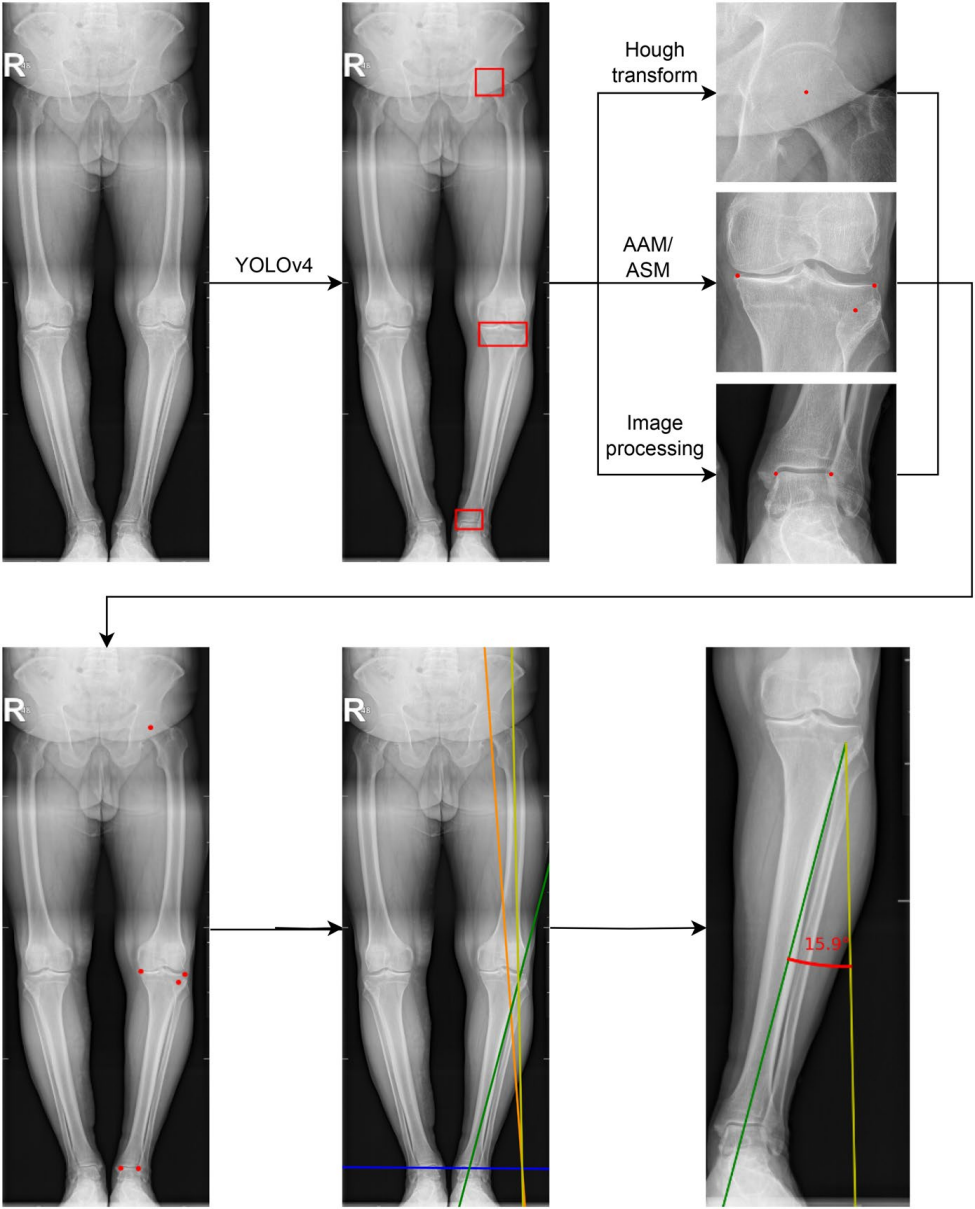
Block diagram of the algorithm for calculating the HTO correction angle using the Miniaci method. The correction angle is the angle between the yellow and green lines.

Figure 2 shows block diagram of our method. First, the image is processed by the YOLO model in order to extract regions of interest: femoral head, knee, and ankle. Then from each region, we detect points according to the Miniaci method. Then by applying analytic geometry formulas we can calculate the estimated correction angle.

Using the datasets mentioned in section “Methods”, we were able to successfully train the YOLO model, achieving a near-perfect mAP score on the validation set, hinting at the possibility of reducing the model complexity. The YOLO model was trained and validated using publicly available datasets and the X-rays of the ankle regions dataset, therefore as a test set for this model we have used the whole long-leg standing X-rays dataset. Of all 54 test images, yielding a total of 108 legs, our YOLO model was unable to detect one bounding box of a femoral head with pathological changes. Considering that six bounding boxes are usually located on each image (two legs, ankle, knee, and femoral head), such a result indicates good model’s performance.

We used the circular Hough transform to detect the center points of the femoral head and image processing methods to determine the inner and outer points of the talus. In the case of knee, experiments were carried out with the Active Shape Model (ASM) and Active Appearance Model (AAM) and that changed the number of landmarks in the training shape. The results are summarized in Table 1 and show that using the AAM model, the final prediction of angles achieves a lower mean, median, and maximum error than the ASM model and that the greater the number of training points, the better the model fits the shape. In the case of the ASM model, we observed that the model is prone to a discrepancy in shape fitting, resulting in high maximum errors of the angle prediction. On the basis of the achieved experimental results, we chose AAM models with 59 training points as the final model to determine the knee points using our automatic method.

**Table 1:** Mean, median and the range of errors (with ICC and 95% CI) in calculating the correction angle with the use of ASM and AAM algorithms to detect knee points for three variants of the number of training points (17, 31, 59) for each of these models. Error was calculated as absolute difference between predicted angle and mean angle from three specialists. Significant values are in bold.

No. training points	Knee points detection algorithm	Median	Mean	Min	Max	ICC (95% CI)	p-value
**59**	**AAM** **left**	**0.3**	**0.5**	**0.02**	**2.76**	**0.99** **(0.97–0.99)**	**5.50E**−**33**
59	ASM left	1	1.2	0	3.97	0.94 (0.9–0.97)	1.98E–20
**59**	**AAM** **right**	** 0.3**	**0.5**	**0**	**1.84**	**0.99** **(0.98–0.99)**	**2.10E**−**34**
59	ASM right	1.2	1.3	0.02	4.36	0.94 (0.9–0.97)	2.19E−21
31	AAM left	0.3	0.6	0.01	3	0.98 (0.96–0.99)	2.38E−30
31	ASM left	1.3	1.5	0.02	4.58	0.92 (0.85–0.96)	2.31E−17
31	AAM right	0.4	0.6	0	2.14	0.99 (0.97–0.99)	4.58E−33
31	ASM right	1.2	1.4	0.01	7.17	0.92 (0.85–0.96)	8.14E−19
17	AAM left	0.4	0.7	0.02	3.66	0.97 (0.95–0.98)	4.74E−26
17	ASM left	1.2	1.4	0.15	3.84	0.92 (0.83–0.96)	5.48E−19
17	AAM right	0.5	0.6	0.02	2.48	0.98 (0.97–0.99)	6.25E−32
17	ASM right	0.7	1.2	0.02	6.52	0.93 (0.88–0.96)	7.77E−20

**Figure 3:**
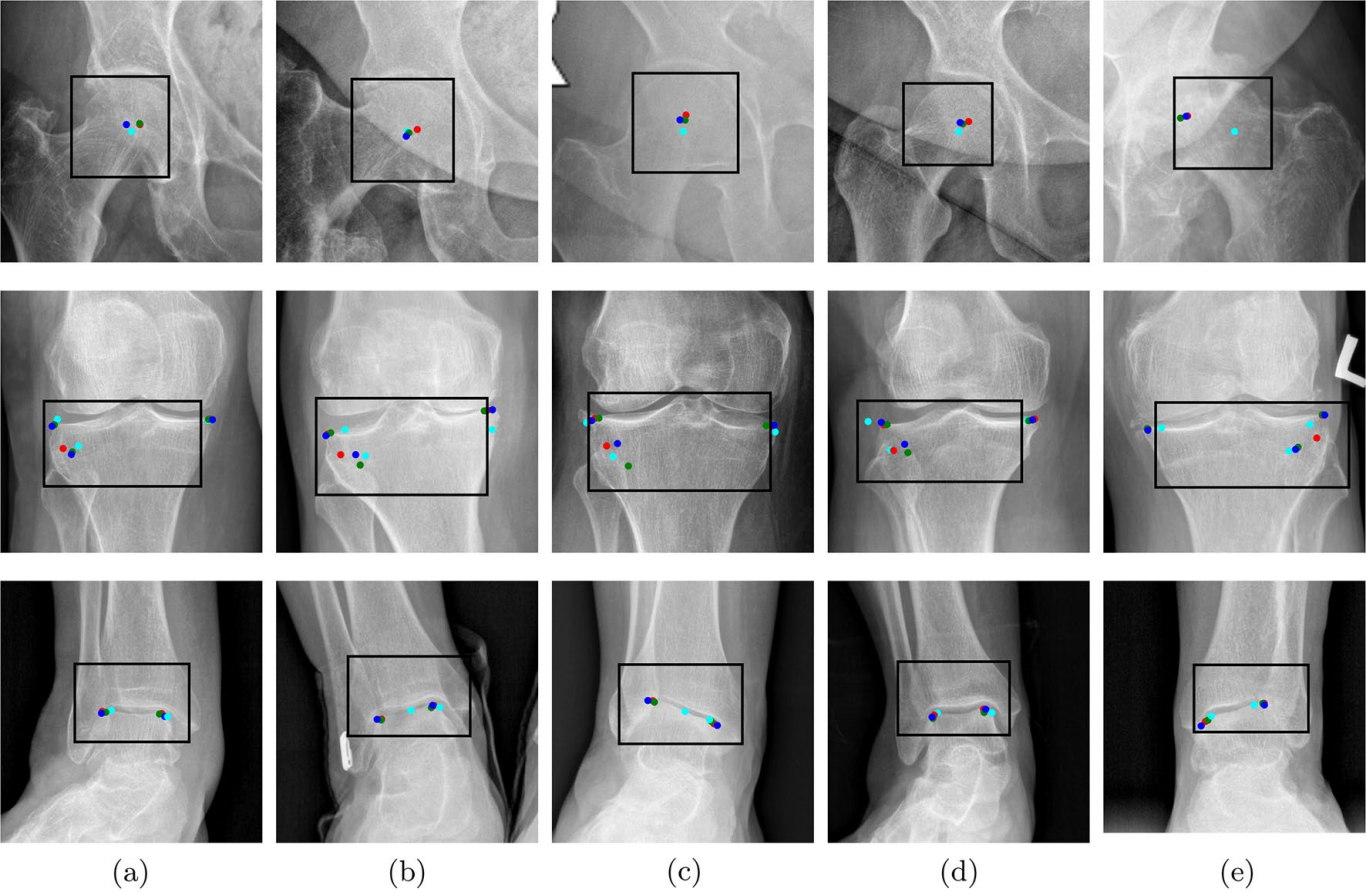
Sample results in a close-up on hip–knee–ankle ROIs. Black frames are the bounding boxes predicted using YOLO, cyan dots are points predicted by our algorithm and red, green, blue dots are points marked by three orthopedic specialists. (**a**) Predicted: 0.64$^{\circ }$, Specialists mean: 0.64$^{\circ }$, Error: 0.00$^{\circ }$. (**b**) Predicted: 24.2$^{\circ }$, Specialists mean: 24.86$^{\circ }$, Error: 0.66$^{\circ }$. (**c**) Predicted: 3.92$^{\circ }$, Specialists mean: 5.18$^{\circ }$, Error: 1.26$^{\circ }$. (**d**) Predicted: 12.96$^{\circ }$, Specialists mean: 11.12$^{\circ }$, Error: 1.84$^{\circ }$. (**e**) Predicted: 8.86$^{\circ }$, Specialists mean: 11.44$^{\circ }$, Error: 2.76$^{\circ }$.

Figure 3 shows a few examples of the results of our fully-automatic method. We selected examples with minimum (Fig. 3a) and maximum (Fig. 3d,e) difference between the predicted angle and the mean angle of specialists. Figure 3b represents the leg for which the correction angle is the highest in our dataset, and Fig. 3c was chosen to show an incorrect detection of the ankle point due to its rotation. In Fig. 3a the points determined automatically and the points determined by specialists coincide and the predicted angle is equal to the mean observer angle, while in Fig. 3b there is a visible rotated ankle and incorrect marking of its external point and shift to the center of the points on the outer part of the knee. However, in the case of advanced varus legs, such errors do not significantly affect the difference in the calculating correction angle. Figure 3c shows that the error resulting from the inaccurate prediction of the points on the rotated ankle is higher. In the case Fig. 3d, the outer knee point was incorrectly predicted. The error in the case of Fig. 3e is influenced by an incorrect bounding box on the femoral head, which is due to its visible pathological deformation and the invisible joint space in the X-ray photo.

**Figure 4:**
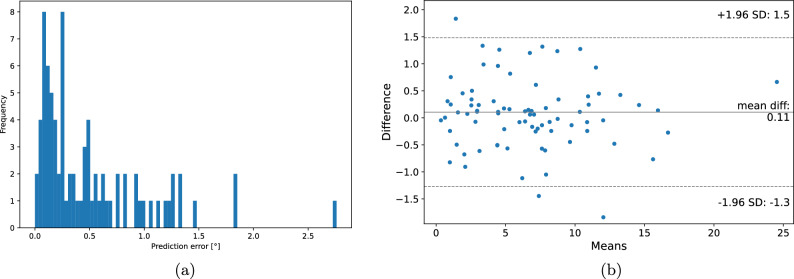
Visualization of algorithm errors in comparison to the mean observer. (**a**) Distribution of errors in automatic determination of the correction angle. The prediction error was calculated as the absolute difference between the predicted angle and the mean observer angle. (**b**) The Bland–Altman plot for mean observer angle and predicted angle.

**Table 2:** Comparison of three raters (R1, R2, R3), mean rater (MR) and fully-automated method (FAM) proposed in this paper. Mean, median and the range of absolute difference errors (with ICC and 95% CI) in calculating the correction angle were calculated.

	Median	Mean	Min	Max	ICC (95% CI)	p-value
R1 vs R2	0.3	0.4	0.01	1.29	1 (0.99–1)	2.25E−83
R1 vs R3	0.3	0.3	0	1.17	1 (0.99–1)	1.01E−85
R2 vs R3	0.3	0.3	0	0.85	1 (0.99–1)	9.57E−90
FAM vs R1	0.3	0.5	0	2.36	0.99 (0.98–0.99)	7.46E−66
FAM vs R2	0.3	0.5	0.02	2.92	0.99 (0.98–0.99)	9.64E−64
FAM vs R3	0.4	0.6	0	3	0.99 (0.98–0.99)	6.16E−64
MR vs R1	0.2	0.2	0	0.74	1 (1–1)	2.29E−102
MR vs R2	0.2	0.2	0.01	0.71	1 (1–1)	1.10E−105
MR vs R3	0.1	0.2	0	0.51	1 (1–1)	1.39E−108
MR vs FAM	0.3	0.5	0	2.76	0.99 (0.98–0.99)	3.12E−66

Table 2 presents the results of the comparison of the correction angles between each of the three orthopedic specialists, the mean observer, and the proposed fully automatic method. The median and mean errors between the algorithm and the raters were slightly higher than the values obtained by the different raters. The maximum differences between the orthopedic specialists were greater than 1$^\circ$. All combinations of assessments obtained a very high ICC coefficient, which means excellent reliability, so these methods can be used interchangeably. Figure 4a shows the distribution of angle errors. Apart from the three outliers (shown and explained in Fig. 3), the error values are less than 1.5$^\circ$ and a large proportion of the errors are below 0.5$^\circ$. The Bland–Altman plot shown in Fig. 4b shows that all points except three lie within the 95% acceptable agreement level. The systematic error is low and indicates that the algorithm is prone to slightly overestimating angles. The analysis of the Bland–Altman plot^14,15^ proves a statistically acceptable agreement level between the mean observer and our fully automated algorithm. According to Jiang et al.^16^ to obtain the correct value of the Hip–Knee–Ankle (HKA) angle after HTO, it is necessary to determine the correction angle using the Miniaci method with an accuracy of ± 1.63$^\circ$. Apart from the three outliers, the error values obtained by our automated method are within the given range. Moreover, examples with outliers have points that are clearly incorrect, so they can be quickly identified and corrected by orthopedist.

## Discussion

High Tibial Osteotomy is performed successfully as a surgical operation to treat knee osteoarthritis and knee with varus deformity. Due to the fact that manual or semi-automatic preoperative planning is time-consuming for doctors, we propose a fully-automatic method for calculating the correction angle using the Miniaci method. For the development and testing of the method, we prepared our own database of 70 long leg standing X-rays performed in an anterior-posterior (AP) view, which is available in conjunction with this article. Our method consisted of several stages: ROI detection using YOLOv4, and then detection of characteristic points using the Hough transform, ASM/AAM and image processing methods. The performance of the ASM and AAM models was compared in different training landmark configurations. Finally, the method of knee point detection with the use of AAM was chosen. The results of our method were compared with three orthopedic specialists using the ICC coefficient and the Bland–Altman plot. Compared to the mean observer, our model obtained the mean absolute error of 0.5$^{\circ }$ and the median of this error was 0.3$^{\circ }$. The ICC ratio was 0.99 (0.98–0.99 95% CI). The highest error was 2.74$^\circ$ and it was due to the incorrect detection of the femoral head by YOLO, which was caused by its deformation. The remaining cases with a high value of absolute error in relation to the average observer most often result from the rotation of the leg in the X-ray image. Incorrectly marked points are clearly visible and can be corrected by the specialist planning the operation. Differences between individual raters, amounting to even 1.29$^\circ$, are also clearly noticeable. However, according to simulation research carried out by Jiang et al.^16^ to obtain the postoperative value of the HKA angle within the physiological range suggested, the correction angle should be determined with an accuracy of ± 1.63$^\circ$. It follows that both the results of our method (except for three outliers) and the results of the orthopedic specialists can be considered consistent with each other and that all methods show a statistically acceptable agreement. The proposed method was successfully tested on the most common correction angles, both those for which HTO surgery is suggested and those that are correct, which may be useful as a part of the screening assessment of the limb axis alignment. One of the disadvantages of our method is that it cannot be applied to the rotated limbs in the photo, which may result from an abnormality in the alignment of the legs.

## Methods

### Datasets

Long-leg standing X-rays performed in an anterior-posterior (AP) view were taken at ORTOTOP Medical Center (Poland)^17^. The entire dataset contained 70 X-rays. All patients agreed to share the anonymized images for research and commercial use. We excluded samples that contained implants, reducing the size of our data set to 54 examples. We divided the dataset into training and test sets in a ratio of 1:3, respectively (13 images in the training set, 41 in the test set).

The femoral head segmentation data set^18^ was used to train the YOLOv4 network, with the bounding boxes of the head automatically marked using the circular Hough transform, with the potential errors manually corrected. For further improvement of the YOLO modelperformance in the femoral head detection task, we used the “Pelvix X-ray images for PelviXNet model” dataset^19^, which contains 150 X-ray images of the pelvises, which we labelled manually.

To detect the knee region of interest, the Knee Osteoarthritis Severity Grading Dataset^20^ was used, which contains 4130 images of both knees per image, of which 200 images were randomly sampled from the data set. Each image was upscaled to four times the original size to ensure that bounding box sizes are consistent with the rest the training data. After the upscaling, we labelled the images manually.

The X-rays of the ankle regions of interest were manually labelled on images provided by the ORTOTOP Medical Center (Poland)^21^; 92 images of the ankle region with potentially large context (e.g., the tibial shaft) were labelled manually.

In order to increase the robustness of the YOLO object detector, the COVID-19 dataset^22^ was used as negative samples. It contains 900 chest X-ray images of COVID-positive cases from which we used 200 samples for our experiments.

### Ethics statement

The data of the long-leg standing X-rays and X-rays of the ankle were provided by Ortotop Medical Center (Poland). This study was non-interventional and retrospective, all participants in the study signed the written informed consent, and the images used in this data were anonymized. A sampled and desensitized example dataset was shared in the data repository. The methods were performed in accordance with relevant guidelines and regulations and approved by Biomedical Commission of the Kraków Medical Chamber (OIL/KBL/1/2023).

### Miniaci method

The open-edge medial high tibial osteotomy (HTO) is one of the procedures used to treat medial knee osteoarthritis. This is an orthopedic procedure performed to restore the mechanical axis of the lower extremity and relieve patient pain.

In order for the procedure to be successful, proper preoperative planning is necessary. In the preoperative planning the Miniaci method is used. First, a mechanical axis of the limb is marked, connecting the center of the femoral head with the center of the ankle joint (MA-mechanical axis). The next line should start from the center of the femoral head and pass through the so-called Fujisawa point at the knee level. The Fujisawa point is 62.5% lateral to the medial edge of the tibial plateau (CA correction axis)^16,23^. Next, a hinge point (HP) has to be assessed. This point is the center of rotation and angulation (CORA). It must be correctly positioned along the tibial plateau and in the reference to the lateral cortex of the tibia. Determining this point is crucial because it is mandatory to achieve stable lateral hinge. Proper hinge point reduces the risk of fracture of the tibial articular surface. However, exact values were not yet estimated, it is assumed that this point should be 20–25 mm below the tibial plateau and 5–10 mm medially from the tibial lateral cortex^13^.

Next, the starting point of the osteotomy is determined in the medial tibial cortex. It should not be marked closer than 30mm from the joint line and not farther than 50–60 mm from it. Finally, the opening correction angle $\alpha$ is determined (correction angle). The first line connects the hinge point (HP) with the center of the ankle joint. The second line connects HP with the intersection of the line that passes through the Fujisawa point at the level of the ground. The angle $\alpha$ formed between these lines is the correction angle^13^.

### Our automatic method

Our method of determining the landmarks to calculate the correction angle according to the Miniaci method consists of several steps:

1. Detect femoral heads, knees, and ankles using YOLO.
2. Detect points within predicted bounding boxes:
  - Find the center of the femoral head using the Hough transform.
  - Find inner and outer knee points using Active Appearance Model (AAM) and Active Shape Model (ASM).
  - Find talus border points using image processing algorithms.
3. Calculate the position of the Hinge point.
4. Calculate the correction angle according to the Miniaci method.

### YOLO

Local shape alignment methods, mentioned below, tend to be significantly divergent when applied to full-scale images without proper shape initialization^24^. For this reason, an object detection method is required to provide a consistent and accurate method for locating the regions of interest. Recently, there have been proposed many methods for locating objects on an image and classifying them, from which we chose the Scaled-YOLOv4-P6 architecture^25^, as it allows to train the model on data sets containing only the regions of interest with some surrounding context, e.g. images of knees, or femur heads only. Due to the availability of pre-trained models for other use cases, it is possible to acquire a well performing model with using as little as a couple of hundred labelled images from data sets that separately do not contain all the regions of interest.

The Scaled-YOLOv4 models internally utilize multiple convolutional layers composed of CSP blocks, first introduced in the CSPNet architecture^26^. CSP blocks are then stacked depth-wise to create a deep neural network. The P6 variant also uses upscaling and downscaling layers on top of the CSP blocks stack. Training such a model requires a data set consisting of pairs of images and labels, where each label contains the classes, positions, and sizes of bounding boxes that designate each of the regions of interest on a given image. In our case, we merged multiple data sets containing regions that were to be detected—the femoral head, lower part of the knee, containing the tibial plateau, and the ankle joint. The model output consists of the bounding boxes located along with the objectness and class probabilities, the objectness score indicating how confident the network is that an object was found, and the class score determining the actual class of the object.

An important feature of the YOLOv4 models family is the ability to predict multiple bounding boxes per image, which are then filtered using a specified objectness score threshold and finally merged using the Non-maximum Suppression algorithm. The two-step approach also allows potential improvements to the model if the recall is small, e.g., it is possible to lower the objectness threshold to increase recall, and use domain-specific algorithms to remove invalid bounding boxes, e.g., removing detected ankle joints with small objectness score when a femoral head was also detected in close proximity. Because in our case the model performance without such improvements was sufficient, we only mention such solutions as a potential improvement in domain-specific applications.

The exact implementation we used is the open-source darknet implementation by Alexey Bochkovskiy^27^.

### Hough transform

We used the Hough transform to detect the femoral head on fragments of photos cropped with the use of bounding boxes provided by YOLO. Finding circles using the Hough transform consists of two steps: detecting edges and finding possible circle centers and then calculating the best radius for each potential circle center. Hough method looks for the best values of parameters, described by Eq. (1).

1 $$\begin{aligned} {(x-a)}^2+{(y-b)}^2 = r^2 \end{aligned}$$

where, *x*, *y*—circle border points; *a*, *b*—circle center points; *r*—circle radius. Then based on the accumulated votes in the multidimensional space, the parameters that are in the peaks of the accumulated votes table are selected. One of the first methods for selecting maximum peaks is the one proposed by Gerig and Klein^28^. The center of the selected circle is in the direction of the maximum gray level gradient for the boundary points of the circle. In our method, the search for circles was limited to the radii of the circle proportional to the size of the bounding boxes. The implementation of the Hough transform algorithm comes from the Scikit-image library^29^.

### Knee points detection

We labelled the training set knee photos—separately for the left leg and the right leg. 57 points for each joint were annotated, using the data labelling software, Label Studio^30^. We also performed experiments with 15 and 29 points labelled for each joint. For each model of points there were characteristic inner and outer knee’s points, determined as the average of the points marked by three specialists added. We conducted experiments to fit the ASM and AAM models on the full leg images by moving the initialization shape to the center of the bounding box predicted by YOLO. We trained the ASM and AAM models on images after performing histogram equalization. To select the hyperparameters of each model, we separated the validation set from the training set (30% of samples) and compared the fitting error in relation to the shape of the ground truth. For the implementation of both models, we used the Menpo Project^31^.

### Active shape model

Active shape model fits the model to the edges of the image. Most often the initializing shape is one of the training shapes. Iteratively, the algorithm evaluates the surroundings of each point to fit the shape to the target object (in our case the tibial plateau). Points are searched along profiles normal to the shape. Most often these points contain the strongest edges along the profile, but may also contain weaker edges or other structural features. During the training, a statistical model of the gray-level structure is built from the search along the normal profiles to the boundary model for each of the points. The effectiveness of fitting the statistical model of the gray-level structure to the new image is assessed using the Mahalanobis distance. We minimize the distance to maximize the probability that the new sample comes from the model distribution^32,33^.

### Active appearance model

Appearance models are generated from a model of shape variation and a model of the appearance variations in the shape-normalized frame. The landmarks are aligned to a common coordinate frame, and then the principal component analysis (PCA) is applied. The estimation of the model parameters, pose, and texture transformation is carried out iteratively until the error reduction is reached and convergence occurs. During model fitting, the points within the image frame are projected onto the texture model frame. Afterwards, the error vector is calculated and the displacements are marked, and the model parameters are updated. In the next step of the iteration new points within the frame texture model are calculated, re-sampling pixels around the given area of the image and evaluating new error vector. If the error is not within the acceptable range, the parameters are updated with a different factor in the next iteration^32,34,35^.

### Hinge point calculation

We determined the Hinge Point based on the labels marked on the training set by the specialists. We calculated the percentage distance of the Hinge Point in the X axis and the Y axis of the image from the outer point of the knee in relation to the width of the knee. The value of the relative shift on the X axis was 14% and on the Y axis 18% of knee width.

### Talus border points determination

We applied the horizontal Sobel filter, thresholding, and morphological transformations such as opening, closing, and removing small objects in order to segment the horizontal part of the tibiotalar joint space. On the binary mask prepared in such a way, the outer points were determined. We used their coordinates to calculate the correction angle according to the Miniaci method. The implementation of the image processing algorithms comes from the Scikit-image library^29^.

### Intra-class correlation coefficient

Correction angle calculation for the high tibial osteotomy depends on the particular orthopedists that mark the points and on the specific method that is used. Therefore, there is no “true” value that can be used to assess the validity of the model. Inter-rater reliability is a metric that allows one to determine the degree of agreement between different observers. Researchers (systems) characterized by high reliability and consistency between evaluators can be used interchangeably^36^. The ICC coefficient is determined in the range of 0–1, where values below 0.5 mean poor reliability, between 0.5 and 0.75 moderate reliability, 0.75 and 0.9 good reliability, and values above 0.9 excellent reliability based on the 95% confidence interval^37,38^. In our case, we measured inter-rater reliability using the intra-class correlation coefficient (ICC). To calculate the ICC, we used the implementation from the Pingouin Python package^39^. To assess the reliability of our method, we asked three independent orthopedic specialists to mark the points on the images needed to determine the correction angle using the Miniaci method. The ICC was determined for the angles calculated based on the points marked by the orthopedists and for the angles calculated with our fully automatic method.

## Data Availability

The data were published as two separate sets: (1) X-ray scalometry images of patients with leg deformity https://data.mendeley.com/datasets/bpvj6xczm2^17^, (2) X-ray ankle joints https://data.mendeley.com/datasets/dskmk9cb8g^21^.

## Code availability

The code is available at https://github.com/medtransfer/high-tibial-osteotomy.
